# Exposure of Sub-adult Nile Crocodiles (*Crocodylus niloticus*) to Extreme Lead Concentrations: A 48-week Experimental Study with Implications for Wild Populations

**DOI:** 10.1007/s00244-025-01159-0

**Published:** 2025-09-24

**Authors:** Fortunate Davhana, Marc Humphries, Gareth Hunter, Nimmi Seoraj-Pillai, Xander Combrink

**Affiliations:** 1 Department of Nature Conservation, Tshwane University of Technology, Staatsartillerie Road, Pretoria, 0183 South Africa; 2 School of Chemistry, University of the Witwatersrand, 1 Jan Smuts Avenue, Braamfontein, Johannesburg, 2050 South Africa; 3 Veterinary Services Department, Johannesburg Zoo, Jan Smuts Avenue, Parkview, Johannesburg, 2000 South Africa

## Abstract

Lead (Pb) poisoning poses a significant threat to wildlife. A primary cause of Pb poisoning is the unintentional ingestion of Pb ammunition and fishing weights, which are still used for hunting and fishing in numerous regions globally. While the effects of Pb poisoning on birds and mammals are well established, impacts on reptiles are less well documented and difficult to assess under field conditions. In this study, we investigated the effects of extreme Pb exposure on captive sub-adult Nile crocodiles (*Crocodylus niloticus*; *n* = 18). We administered Pb dosages in the form of fishing weights (54–215 g) and monitored changes in blood lead concentrations, packed cell volumes, urine Pb concentrations, growth, and body condition over a 48-week period. Crocodiles exhibited a remarkable tolerance to exceptionally high Pb exposure over the duration of the study. Despite the lack of obvious clinical signs of Pb toxicity, elevated BPb concentrations were linked to lower PCVs, indicating anaemia across all treatment groups by week eight. However, crocodiles showed a sustained erythropoietic response which may be contributing to their resilience to acute Pb toxicity. While Pb exposure did not significantly affect body condition, it was associated with a discernible reduction in weight gain over the duration of the study. Our estimation of a 5.8–7.3-year timeframe for complete dissolution of the Pb fishing weights in the experimental crocodiles’ stomachs carries significant implications for wild populations, which are likely to be exposed to Pb for far longer than 48-week duration of this study.

## Introduction

Lead is an inessential heavy metal that is ubiquitous in the environment and poses significant threats to humans and wildlife globally (Buekers et al. 2009; Pain et al. 2019). Both acute and chronic exposure to Pb is linked to numerous deleterious effects, including disorders of the nervous, gastrointestinal, circulatory and reproductive systems (Vallverdú-Coll et al. 2019; Monclus et al. 2020). Although wildlife can be exposed to environmental contaminants through inhalation or skin absorption, severe cases of Pb poisoning typically occur through ingestion of Pb objects, such as spent ammunition, fishing weights and Pb-weighted artificial fishing lures (Lance et al. 2006; Warner et al. 2016; Descalzo et al. 2021; Chiverton et al. 2022; Humphries et al. 2022).

Lead poisoning has been most frequently documented in birds, especially waterfowl and raptors (e.g. Pain et al. 2019; Monclus et al. 2020). In waterfowl, ingestion of Pb shot (small spherical pellets loaded into shotgun shells), often mistaken for food or grit, represents a significant route of primary exposure (Green et al. 2016; Pain et al. 2019; Sileo et al. 2001; Green et al. 2022) and is considered one of the leading causes of wildfowl mortality globally (Williams et al. 2018; Pain et al. 2019). In scavenging and predatory birds, incidences of Pb poisoning almost always result from the ingestion of shot or bullet fragments embedded in the tissues of animals killed with Pb-based ammunition (Finkelstein et al. 2012; Pain et al. 2019). Upon ingestion, Pb undergoes dissolution by gastric acids in the stomach, facilitating rapid absorption into the bloodstream and subsequent distribution to organs, ultimately accumulating in bone (Franson and Pain 2011).

Ammunition and fishing weights deposited in terrestrial and aquatic environments also affect non-avian wildlife, although the prevalence of Pb exposure in other taxa is less well documented. Nevertheless, recent reports indicate that Pb exposure is a relevant concern in mammals, with elevated Pb concentrations observed in several species, including brown bears (*Ursus arctos*; Fuchs et al. 2021; Brown et al. 2024), grey wolves (*Canis lupus*; Kelly et al. 2021), and Tasmanian devils (*Sarcophilus harrisii*; Jones et al. 2024). Elevated Pb concentrations have also been reported in reptiles. These include clinical cases in snapping turtle (*Chelydra serpentina*; Borkowski 1997) and red-eared terrapin (*Trachemys scripta elegans*; Jimenez & Martinez 2002). Lead poisoning has also been documented in crocodilians. Camus et al. (1998) and Lance et al. (2006) documented Pb poisoning in captive American alligators (*Alligator mississippiensis*) fed minced nutria (*Myocastor coypus*) contaminated with Pb bullet fragments. In Kakadu National Park, Northern Australia, Twining et al. (1999) found elevated Pb levels in the osteoderms of saltwater crocodiles (*Crocodylus porosus*) inhabiting a heavily hunted region where indigenous people utilised Pb ammunition.

More recently, studies have revealed exceptionally high blood Pb (BPb) concentrations in Nile crocodiles (*Crocodylus niloticus*) from Lake St Lucia, South Africa, an area with a history of intensive recreational fishing (Warner et al. 2016; Humphries et al. 2022). Humphries et al. (2022) documented BPb concentrations of 13,100 ng/ml in St Lucia crocodiles, representing some of the highest levels ever recorded in a free-ranging vertebrate. Fishing sinker (weight) ingestion has been confirmed as the major pathway of Pb exposure in Nile crocodiles at Lake St Lucia, with a post-mortem examination revealing multiple sinkers and Pb-weighted artificial fishing lures within the stomach of a crocodile (Warner et al. 2016). Although crocodilians are generally considered to exhibit a relatively high degree of resistance to environmental contaminants (e.g. Grillitsch and Schiesari 2010), Humphries et al. (2022) identified clinical evidence of Pb toxicosis in St Lucia crocodiles exposed to high levels of Pb. In this study, chronic Pb exposure appeared to be associated with anaemia and severe deterioration in tooth condition, and in one instance, mortality was attributed to Pb poisoning. These findings highlight concerns for the long-term health of the crocodile population at Lake St Lucia as well as other crocodilian habitats impacted by fishing activities.

Field studies investigating the impact of Pb on crocodilians face several inherent limitations. These include challenges in acquiring adequate sample sizes and difficulties in documenting less overt symptoms, such as weight loss and behavioural changes. Thus, despite multiple studies indicating significant Pb exposure in crocodilian populations, impacts on growth and mortality remain unclear. Furthermore, the retention time of ingested foreign Pb objects within the crocodilian stomach, alongside the underlying physiological mechanisms of Pb processing, storage, and resistance to acute toxicity, are still poorly understood. Only a single experimental study to date has investigated Pb exposure in crocodilians. In this study, Hammerton et al. (2003) administered Pb shot to four sub-adult saltwater crocodiles and monitored BPb concentrations over a 20-week period. A significant increase in BPb levels within the first week post-ingestion was observed, followed by a period of stabilisation. Notably, the crocodiles maintained an apparently healthy physical condition and did not exhibit typical clinical signs of Pb poisoning. While Hammerton et al. (2003) interpreted their findings as indicative of a high resistance to Pb toxicity in crocodilians, relatively low dosages were used (1.92–3.76 g Pb) and the 20-week experimental period did not allow for the assessment of potential long-term consequences associated with Pb exposure.

Lead remains in use for hunting and fishing in numerous regions globally. Many of these areas, including Australia, southern Africa, southeastern USA, and Central America, are also home to significant crocodilian populations. Consequently, further research is essential to fully understand the effects of Pb exposure on crocodilian populations. In this study, we investigated the effects of extreme Pb exposure on sub-adult Nile crocodiles. We administered varying doses of Pb, up to 215 g in the form of fishing weights, and monitored changes in BPb, packed cell volume (PCV), and urinary Pb concentrations and assessed red blood cell morphology over a 48-week period. Additionally, we assessed the impact of Pb on growth and body condition. The study represents the longest Pb dosage experiment ever conducted on a reptile and provides unique insights into crocodilian ecotoxicology.

## Materials and Methods

### Experimental design

The study population consisted of 22 randomly selected sub-adult Nile crocodiles (all < 2.5 m total length at the start of the study) on a commercial crocodile farm (Croc Skin Traders, Limpopo Province, South Africa). Only sub-adults were available for research purposes from the farm, and these were therefore used in the study. The crocodiles were all hatched and raised at the same breeding facility where this study took place. Throughout the study period, all crocodiles were housed individually in single enclosures and remained in the same pens until the end of the experimental period. Each enclosure was assigned a unique identification number, which was used to track and record data for the corresponding animal throughout the study. The enclosures (2.4 m × 1.2) consisted of a waterbody sufficiently deep for full submersion as well as a dry emergent area for basking.

The study population was divided into three Pb treatment groups and a control group (Table 1). To mimic field conditions, the Pb dosage was administered using a varying number of two-ounce Pb fishing sinkers. The rational for selecting a two-ounce fishing sinker is the popularity of using this weight in the St Lucia estuarine system. Fishing sinkers used in estuaries are typically lighter compared to heavier ones required (e.g. 3–7 oz) for rock and surf fishing. Lead fishing weights were pre-weighed using an analytical balance and packaged inside one-week-old broiler chicken carcasses. On day zero, the first day of the study, these carcasses were fed to the crocodiles according to their assigned treatment groups. Unconsumed chicken carcasses were retrieved, and the weights repackaged into new chicken carcasses and presented to the same individuals the following day, until all Pb weights had been ingested.

**Table 1:** Administered Pb dosages for experimental treatment and control groups

Group	Number of individuals*	Pb dosage
Treatment 1	5	2 × 1 oz sinkers (~ 54 g)
Treatment 2	3	5 × 1 oz sinkers (~ 135 g)
Treatment 3	6	8 × 1 oz sinkers (~ 215 g)
Control	4	Zero

*The study initially involved 22 individuals. However, post-mortem examination revealed that four individuals had ingested fewer Pb weights than expected for their assigned treatment groups, and therefore, they were excluded from the analysis

**Fig. 1:**
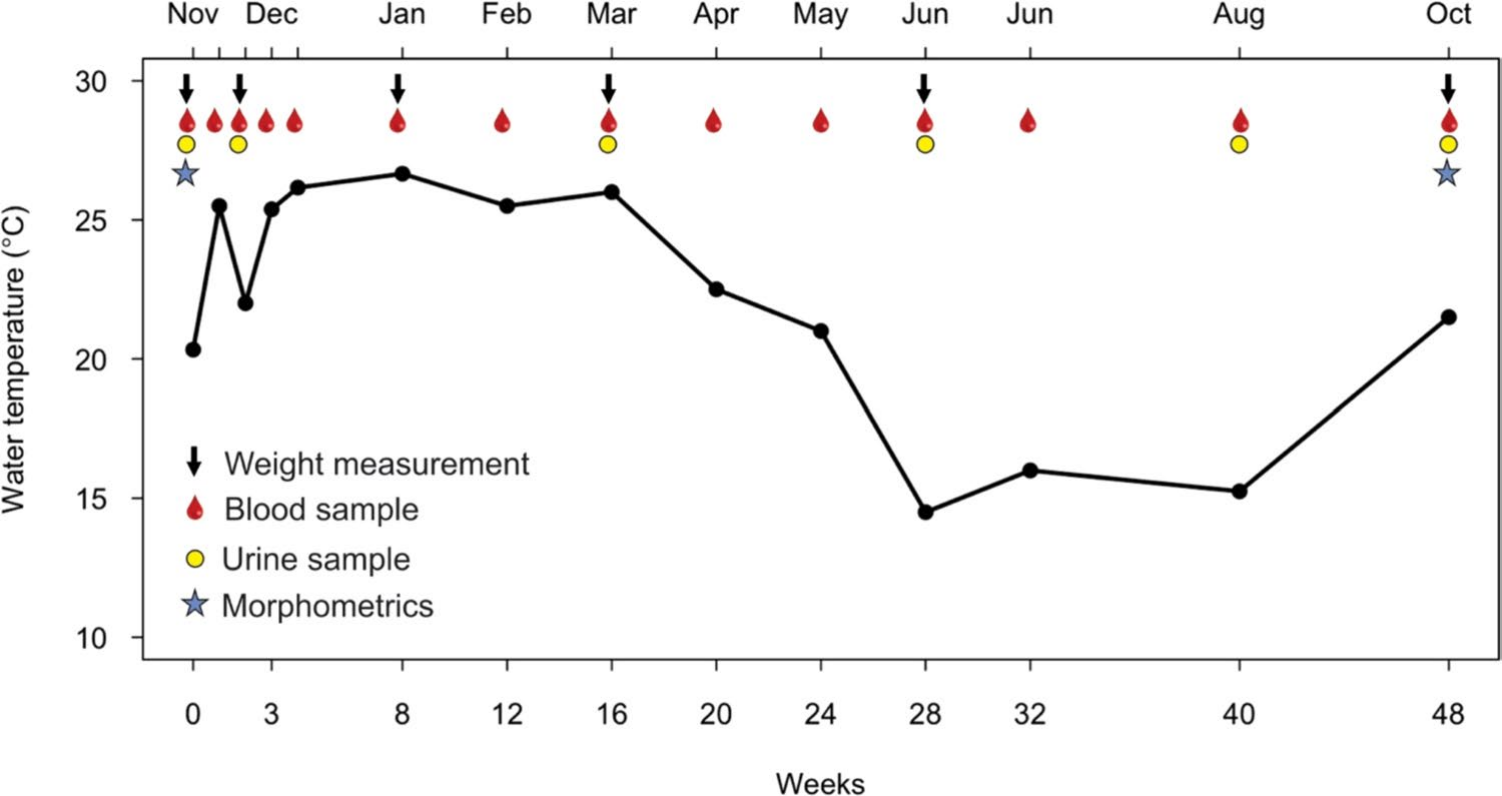
Sampling and monitoring protocol adopted over the 48-week experiment. The red line shows variation in water temperature (°C) within crocodile enclosures over the experimental period

Blood and urine samples were collected at regular intervals over a 48-week period from 15 November 2023 to 24 October 2024 (Fig. 1). Body weight was tracked periodically, with morphometric measurements performed at the beginning and end of the study. This included measurements of total length (TL), snout–vent length (SVL), head length (HL), neck girth (NG), mid-body girth (MBG), and tail girth (TG). Water, air temperature, and humidity were recorded during each sampling session.

### Blood collection and analysis

Blood samples (~ 10 ml) were collected from the post-occipital venous sinus using an 18-gauge hypodermic needle following the procedure described by Myburgh et al. (2014). Blood was sampled once per week for the first month and thereafter once every month until the end of the study. Approximately, 6 ml were transferred to sterile Vacucare lithium heparin tubes (Symbiolab, Johannesburg, South Africa) and transported to the University of the Witwatersrand for Pb analysis. The remainder of the blood (~ 4 ml) was centrifuged, and plasma separated and stored for future biochemistry analyses.

Lead analysis was carried out following previously established protocols (Humphries et al. 2022). Approximately 0.5 g of whole blood was gravimetrically transferred into a pre-cleaned Teflon digestion vessel and treated with 3 ml nitric acid (65% Suprapur, Sigma Aldrich). Digestion was conducted using a Multiwave GO Plus (Anton Paar, Graz, Austria) microwave digestion system. Lead concentrations were measured by inductively coupled plasma mass spectroscopy (Agilent 7700 ICP-MS) equipped with a standard sample introduction system and SPS4 autosampler. Lead concentrations were determined against standard solutions prepared by gravimetric dilution from certified primary elemental solutions. Full procedural blanks were analysed with each batch of samples to monitor for background contamination. The sample digestion and analysis procedures were validated using certified reference material ERM-CE278k (muscle tissue) purchased from Merck, Germany. Analytical results showed good agreement with recoveries averaging 2.21 ± 0.34 mg/kg (*n* = 3; certified value = 2.18 ± 0.18 mg/kg). The detection limit for Pb in water was 0.4 ng/ml.

PCVs (haematocrit) were measured at each sampling occasion using the microhaematocrit method. Whole blood collected in heparinised capillary tubes was spun in a microhaematocrit centrifuge (Digisystem Laboratory Instruments Inc., New Taipei City, Taiwan) at 12,000*g* for 3 min, following the procedures outlined by Weiser (2022).

### Blood smear preparation and analysis

Blood smears were prepared using the coverslip to slide technique described by Perpiñán et al. (2006). These were made from whole blood shortly after collection whenever possible. Otherwise, blood smears were made as soon as possible after sampling from heparinised blood. As part of a preliminary assessment, smears prepared at weeks eight and 48 were examined for signs of red blood cell (RBC) regeneration. Regeneration was assessed by evaluating RBC size, nuclei size, and the degree of polychromasia. The percentages of large RBCs and those with large nuclei were calculated by assessing all RBCs in 5 randomly selected high power microscope fields in the monolayer area of the smear. The percentage of RBCs displaying prominent basophilic stippling was calculated in the same way. Polychromasia was categorised subjectively as normal, mildly increased, moderately increased, or severely increased based on the relative differences in cytoplasmic staining.

### Urine collection and analysis

Urine was generally sampled every alternate month using a urinary catheter (FG 10; 3.3 mm × 500 mm, SMI AG Steinerberg 8, Belgium) following the procedure described by Myburgh et al. (2012). Each urine sample was transferred to a 50-ml tube and transported on ice to the laboratory for Pb analysis. Urine samples were passed through 0.45 µm syringe filters to remove urates and then analysed directly by ICP-MS as described above.

### Weight and morphometric measurements

Body condition was assessed at the start and end of the study by recording the following morphometric measurements: total length (TL), snout–ventral length (SVL), head length, weight, neck girth, mid-body girth, and tail girth. A body condition index (BCI) was calculated for week 0 (start of the study) and week 48 (end of the study) using the formula: BCI = Body mass (kg)/[Total length (cm)]^3^.

In addition, crocodiles were weighed intermittently over the duration of the study using a 60-kg digital hanging scale (Micro Crane Scale, model CS-60 kg, Gauteng, South Africa). Weight measurements were recorded in week 0 (15 Nov 2023), week 3 (9 Dec 2023), week 8 (13 Jan 2024), week 16 (9 Mar 2024), week 32 (29 Jun 2024), and week 48 (22 Oct 2024).

### Calculation of Pb weight erosion

At the end of the study, crocodiles were humanely euthanised according to the “stun and cut method” (SANS 2009; Manolis and Webb 2016). Lead weights retrieved from the stomachs of crocodiles were washed, dried, and then reweighed to calculate the loss in Pb. Erosion rates were calculated as the difference between the total initial and final weight of the Pb sinkers divided by the exposure period (345 days).

### Statistical analysis

All statistical analyses were performed using R software version 4.2.3. (R Core Team 2023), which is freely available from the R Project website: https://www.r-project.org/. The distribution of the data was assessed for normality visually using Q-Q plots and statistically using the Shapiro–Wilk test (Shapiro and Wilk 1965). In cases where the data were normally distributed, one-way ANOVA or paired *t* tests were used. For data that exhibited non-normality even after log transformation, non-parametric analyses, such as Kruskal–Wallis (Kruskal and Wallis 1952) or Wilcoxon signed-rank (Wilcoxon 1945) test were applied. Post-hoc comparisons were conducted using Tukey’s test for ANOVA (Tukey 1949) and Dunn’s test (Dunn 1964) for Kruskal–Wallis. Statistical significance was set at *p* < 0.05. Graphical representations of the data were generated using R, with points representing mean and error bars representing standard error of the mean.

### Ethical approval

This study was approved by the Tshwane University of Technology Animals Research Ethics Committee (AREC202209001). Research approval in terms of section 20 of the Animal Diseases Act was obtained from the Department of Agriculture, Land Reform and Rural Development (12/11/1/1/14; 2963PM), and registered with the Department of Forestry, Fisheries and the Environment (TOPS permit:S-65778).

## Results

### Blood lead concentrations over time

**Fig. 2:**
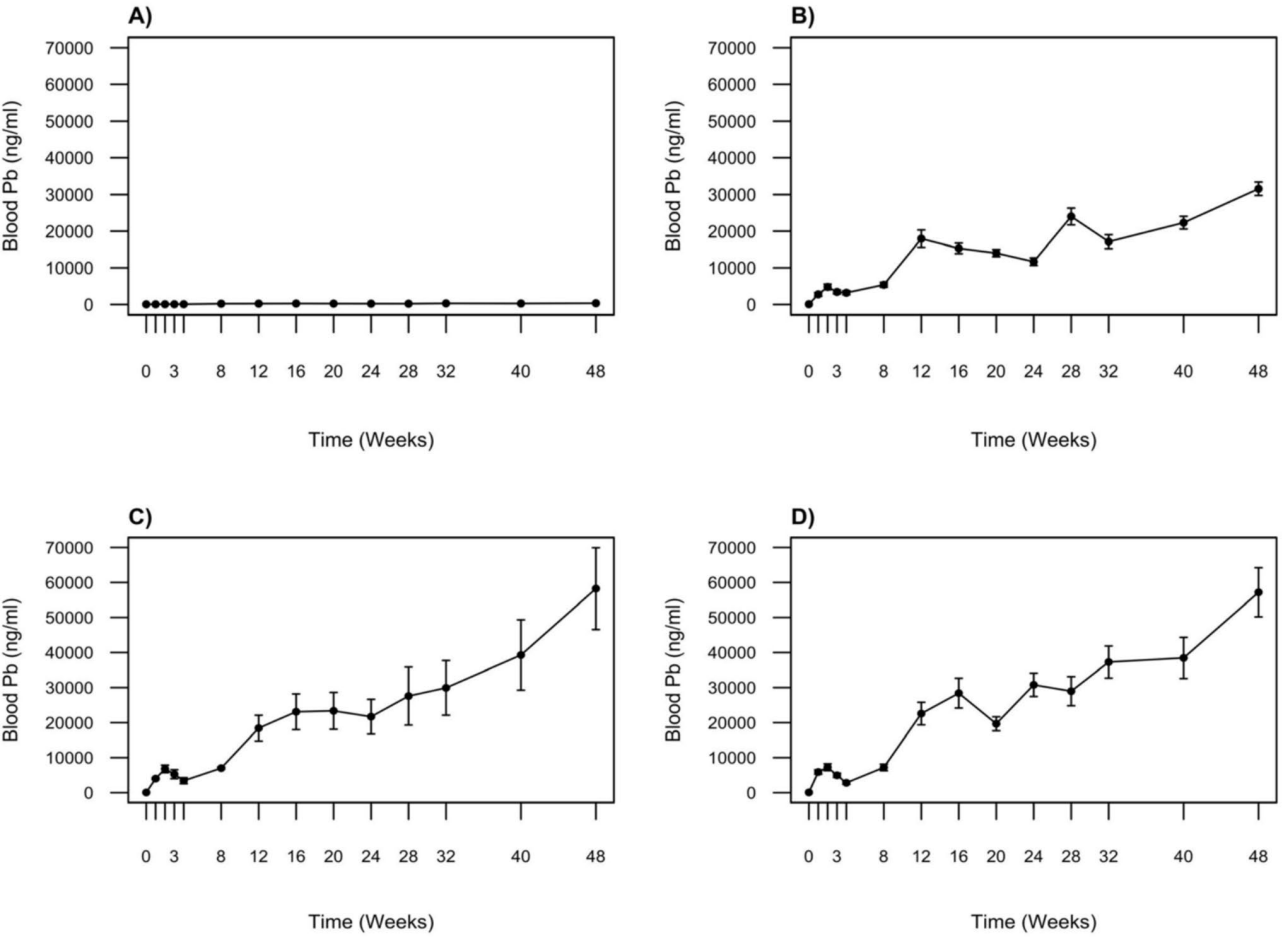
Mean blood Pb (BPb) concentrations (ng/ml) for **A** Control: no lead (Pb) exposure (*n* = 4); **B** Treatment 1: 2 × 1 oz sinkers (~ 54 g Pb, *n* = 5); **C** Treatment 2: 5 × 1 oz sinkers (~ 135 g Pb, *n* = 3); **D** Treatment 3: 8 × 1 oz sinkers (~ 215 g Pb, *n* = 6) over the duration of the study. Data are presented as mean ± standard error of the mean (SEM)

All crocodiles showed low BPb concentrations (60.8 ± 34.4 ng/ml) at the beginning of the study (week 0: Control 42.9 ± 6.3 ng/ml; treatment 1: 73.9 ± 19.6 ng/ml; treatment 2: 53.4 ± 13.2 ng/ml; treatment 3: 65.6 ± 16.8 ng/ml; Fig. 2). Treatment crocodiles showed a rapid increase in BPb concentrations during the first two weeks after exposure to Pb sinkers. By week 2, concentrations had risen to 4390 ± 1150 ng/ml in treatment 1, 6790 ± 1080 ng/ml in treatment 2, and 6420 ± 1550 ng/ml in treatment 3, while the control group remained low (68.1 ± 11.3 ng/ml). The magnitude of increase across the three treatment groups varied in proportion to dosage. In the first week, mean BPb of crocodiles in treatment 3 increased almost 100-fold, while an ~ 40-fold increase was observed in those in the lowest dosage group (treatment 1). During weeks 3 and 4, BPb concentrations appeared to stabilise, and in some cases, slight decreases were recorded. However, BPb concentrations began to increase again across all treatment groups by week 8, reaching high levels by week 24 (treatment 1: 11,700 ± 1040 ng/ml; treatment 2: 21,700 ± 4950 ng/ml; treatment 3: 30,800 ± 3320 ng/ml).

Despite some individual variability within groups, this general upward trend continued until week 48, when maximum BPb concentrations were recorded. There were significant differences in BPb concentrations between treatment groups at the end of the study (*χ*^2^ = 9.0, *p* = 0.011). Treatment groups 2 and 3 had significantly higher BPb concentrations than treatment 1 (*p* = 0.022 and *p* = 0.0057, respectively), while there are no significant differences between treatment groups 2 and 3 (*p* = 1.00). Over the duration of the experiment, mean BPb concentrations in treatment groups 2 and 3 increased approximately 1000-fold, reaching 58,200 ng/ml and 57,200 ng/ml, respectively. A maximum BPb concentration of 31,600 ng/ml was recorded for treatment group 1. Blood Pb concentrations in the control crocodiles remained consistently low throughout the experiment. However, a gradual increase in BPb was observed in all control animals over the duration of the study, reaching a mean of 345 ± 36.8 ng/ml at week 48.

### Packed cell volumes over time

**Fig. 3:**
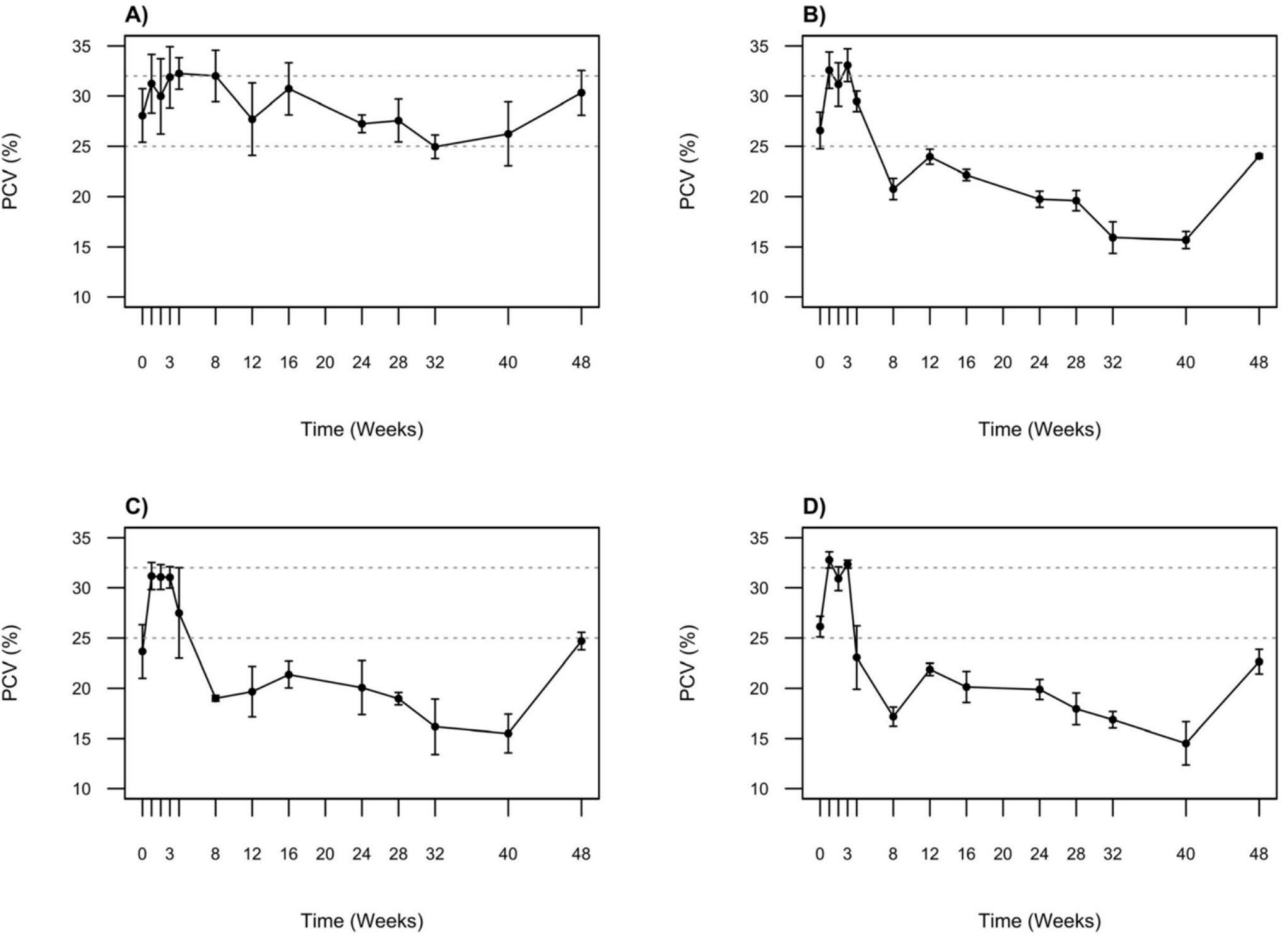
Mean packed cell volume (PCV) for **A** Control: no Pb exposure (*n* = 4); **B** Treatment 1: 2 × 1 oz sinkers (~ 54 g Pb, *n* = 5); **C** Treatment 2: 5 × 1 oz sinkers (~ 135 g Pb, *n* = 3); **D** Treatment 3: 8 × 1 oz sinkers (~ 215 g Pb, *n* = 6) over the duration of the study. The grey dotted lines indicate the reference range established for this study population based on the average upper and lower limits measured in the control group. Data are presented as mean ± standard error of the mean (SEM)

PCV values in control crocodiles varied both among individuals and throughout the duration of the study (Fig. 3). Nevertheless, the mean PCV for this group remained within a relatively range, fluctuating between 32.3 ± 3.2% and 25.0 ± 2.4%. In comparison, the PCVs of treatment group crocodiles showed considerably more variation. PCVs decreased dramatically at week eight across all treatment groups, with lowest values 17.2 ± 2.4% recorded in the highest dosage group (treatment 3). A gradual decrease in PCVs was observed in all treatment groups over the next 32 weeks, reaching a minimum in week 40. This decline was most notable in treatment group 3, where PCVs reached a minimum of 14.5 ± 5.3%. PCVs increased for all treatment groups over the last eight weeks of the study but remained lower relative to the control group.

Kruskal–Wallis tests showed significant differences in PCV between groups at weeks eight, 16, 24, 32, and 48 (*p* < 0.05). Post-hoc Dunn’s tests revealed significant differences between the control group and treatment 3 group at weeks eight (*p* = 0.005), 16 (*p* = 0.031), 24 (*p* = 0.040), and 48 (*p* = 0.026). A significant difference was also observed between control group and treatment group 1 at week 24 (*p* = 0.040) and week 32 (*p* = 0.041).

Red blood cell regeneration.

**Fig. 4:**
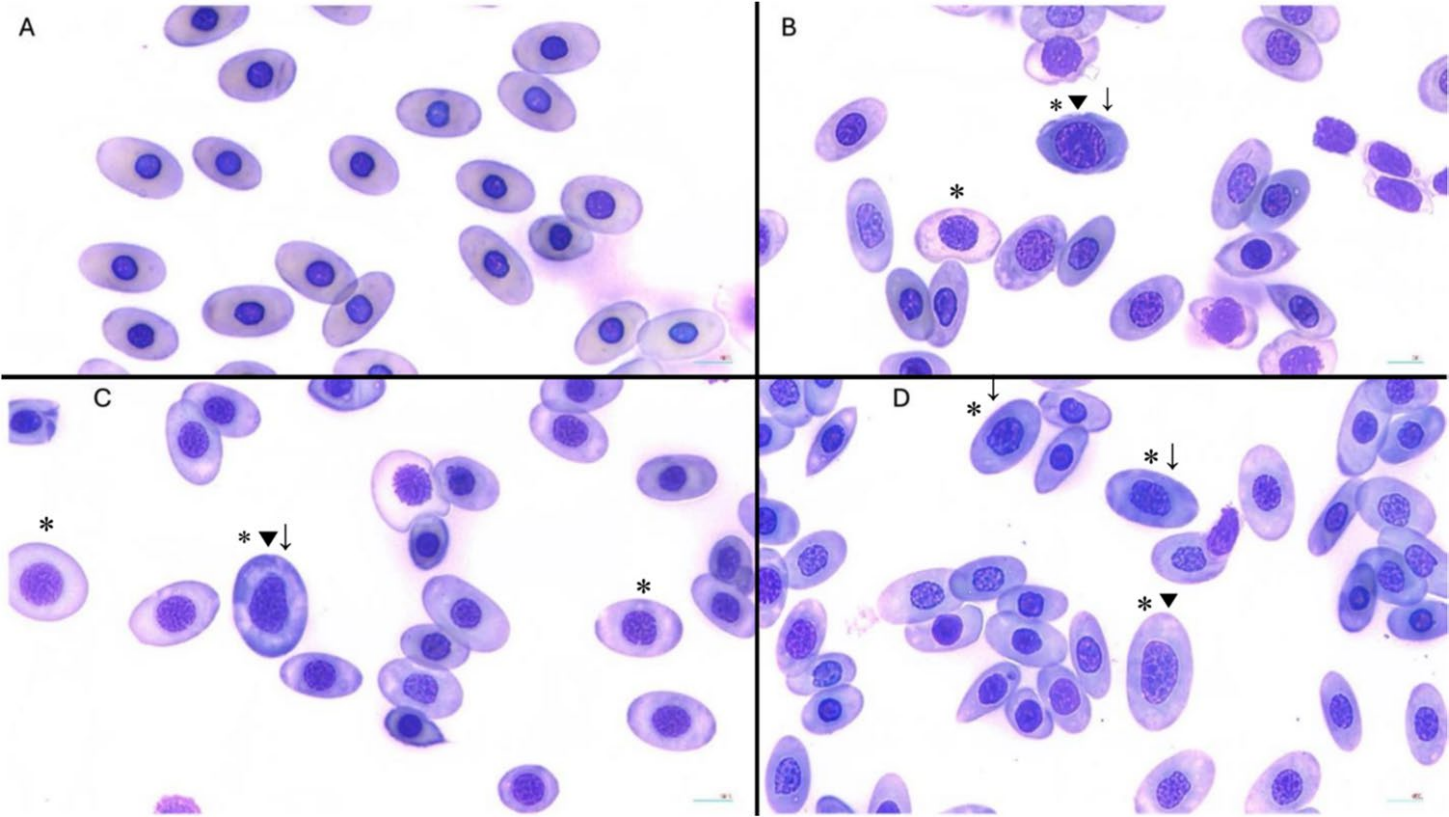
Marked signs of erythrocyte regeneration at week 48 in treatment groups compared to controls. Note the variation in nuclei and overall cell size and cytoplasm colour of the erythrocytes. Cells with enlarged nuclei (*), large in overall size (▼) and having a darker staining cytoplasm (↓) are noted. Cells often had more than one of these characteristics. **A** Control: no lead (Pb) exposure; **B** Treatment 1: 2 × 1 oz sinkers (~ 54 g Pb); **C** Treatment 2: 5 × 1 oz sinkers (~ 135 g Pb); **D** Treatment 3: 8 × 1 oz sinkers (~ 215 g Pb)

**Fig. 5:**
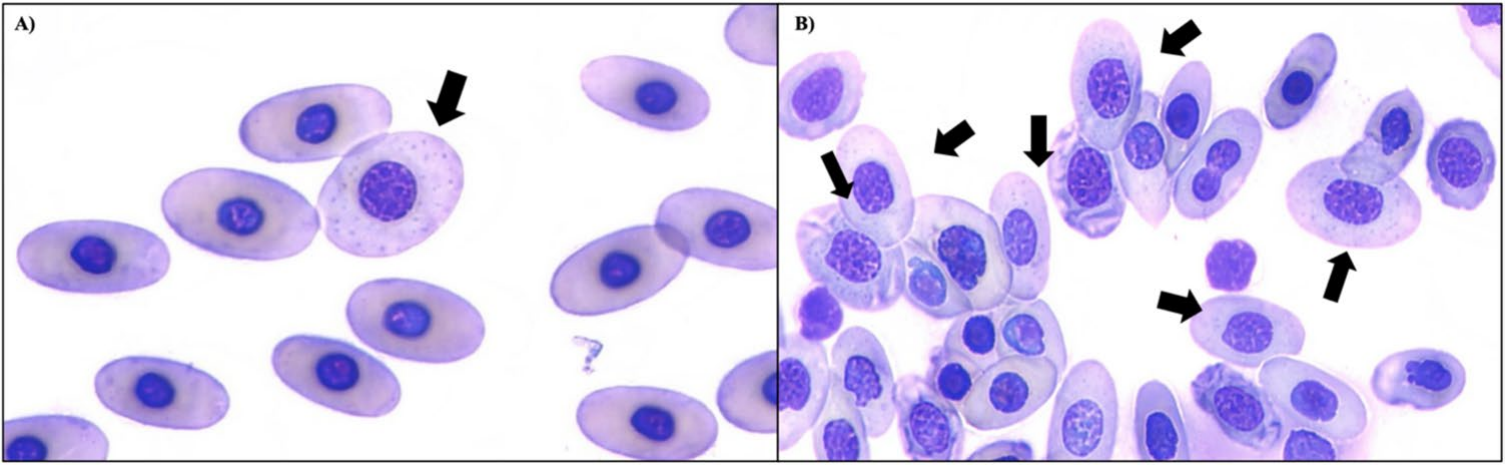
Basophilic stippling in RBCs (arrows) was more commonly seen in crocodiles dosed with lead than in the control crocodiles. This likely reflects aggregates of ribosomal RNA. **A** Control: no lead (Pb) exposure at week 48; **B** Treatment 3: 8 × 1 oz sinkers (~ 215 g Pb) at week 48

All treatment group crocodiles showed signs of increased RBC regeneration compared to the control group, as observed on blood smears (Fig. 4). This included increased polychromasia at both weeks eight and 48 (mild to moderately increased vs. normal in the control crocodiles). A higher proportion of enlarged RBCs was also recorded in treatment groups at week eight (T1: 75.4 ± 13.1%, T2: 87.4 ± 5.8%, T3: 83.8 ± 6.6%, control: 4.9 ± 2.4%) and at week 48 (T1: 88.6 ± 0.6%, T2: 89.0 ± 2.3%, T3: 85.2 ± 7.2%, control: 4.3 ± 0.8%). Similarly, the percentage of RBCs with large nuclei was consistently higher in treatment animals compared to controls at week 8 (T1: 64.8 ± 14.3%, T2: 83.6 ± 7.0%, T3: 78.8 ± 5.0%, control: 6.8 ± 5.0%) and week 48 (T1: 80.4 ± 9.2%, T2: 82.5 ± 2.5%, T3: 80.5 ± 7.3%, control: 9.4 ± 2.2%). These findings indicate a higher percentage of immature RBCs in crocodiles dosed with Pb. Additionally, basophilic stippling was observed more frequently in treatment group crocodiles than in controls (week 8: 1.7 ± 1.9% to 15.1 ± 9.6% in treatment animals vs. none in controls; week 48: 11.0 ± 6.0% to 33.5 ± 15.1% in treatment animals vs. 0.9 ± 0.8% in controls) (Fig. 5).

### Urine lead (UPb) concentrations over time

**Fig. 6:**
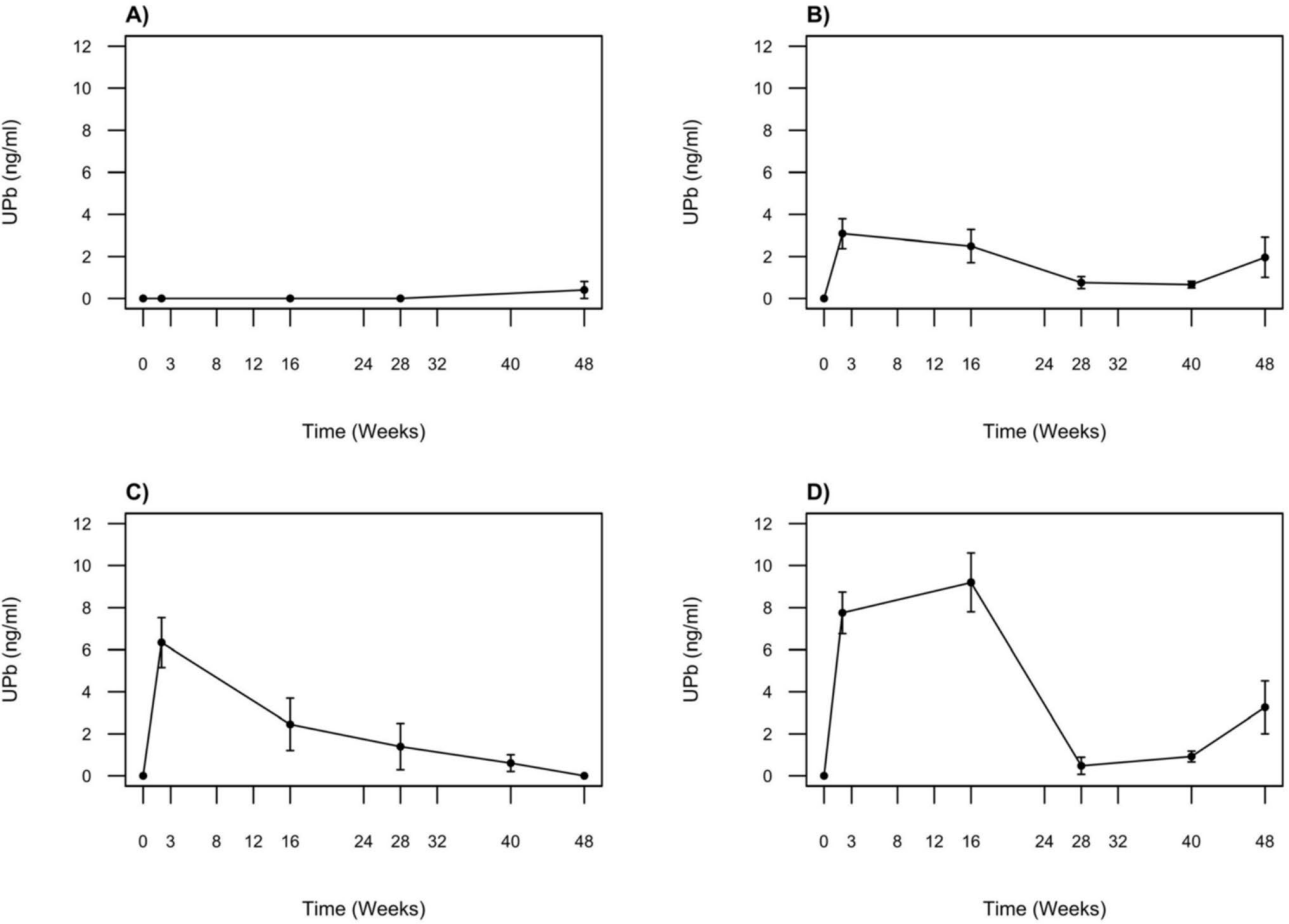
Mean urine lead (UPb) concentrations (ng/ml) in response to different lead (Pb) exposures **A** Control: no Pb exposure (*n* = 4); **B** Treatment 1: 2 × 1 oz sinkers (~ 54 g Pb, *n* = 5); **C** Treatment 2: 5 × 1 oz sinkers (~ 135 g Pb, *n* = 3); **D** Treatment 3: 8 × 1 oz sinkers (~ 215 g Pb, *n* = 6). Data are presented as mean ± standard error of the mean (SEM)

Relatively low concentrations of Pb were detected in urine samples collected over the duration of the study (Fig. 6). Urinary Pb concentrations in control crocodiles fell below analytical detection limits throughout the study, apart from one individual which had UPb value of 2.4 ng/ml at week 48. In the treatment groups, UPb concentrations showed a significant increase (Wilcoxon signed-rank test: *V* = 105.0, *p* < 0.001) during the initial two weeks of the study. By week 2, mean ± SD concentrations were treatment 1: 3.1 ± 1.6 ng/ml, treatment 2: 6.3 ± 2.1 ng/ml, treatment 3: 7.0 ± 2.7 ng/ml. Subsequently, UPb concentrations in treatment groups 2 and 3 gradually decreased over time. Notably, treatment group 3 exhibited the highest UPb concentrations at 16 weeks, with a maximum recorded value of 13 ng/ml and a mean of 9.6 ± 2.9 ng/mL (compared with treatment 1: 2.5 ± 1.8 ng/ml; treatment 2: 2.5 ± 2.2 ng/ml). At week 48, UPb concentrations had decreased to treatment 1: 2.0 ± 2.1 ng/ml, treatment 2: 0.0 ± 0.0 ng/mL, and treatment 3: 3.7 ± 2.8 ng/ml. Maximum UPb concentrations varied with Pb dosage, but did not follow the same trends observed in BPb. Kruskal–Wallis tests showed significant differences in urine Pb concentrations between groups at week 2 (*p* = 0.0299) and week 16 (*p* = 0.0140). Post-hoc Dunn’s tests revealed significant differences between treatment 1 and treatment 3 at week 2 (*p* = 0.029) and week 16 (*p* = 0.016). No significant differences were observed at weeks 28, 40, or 48.

### Lead weight erosion

**Fig. 7:**
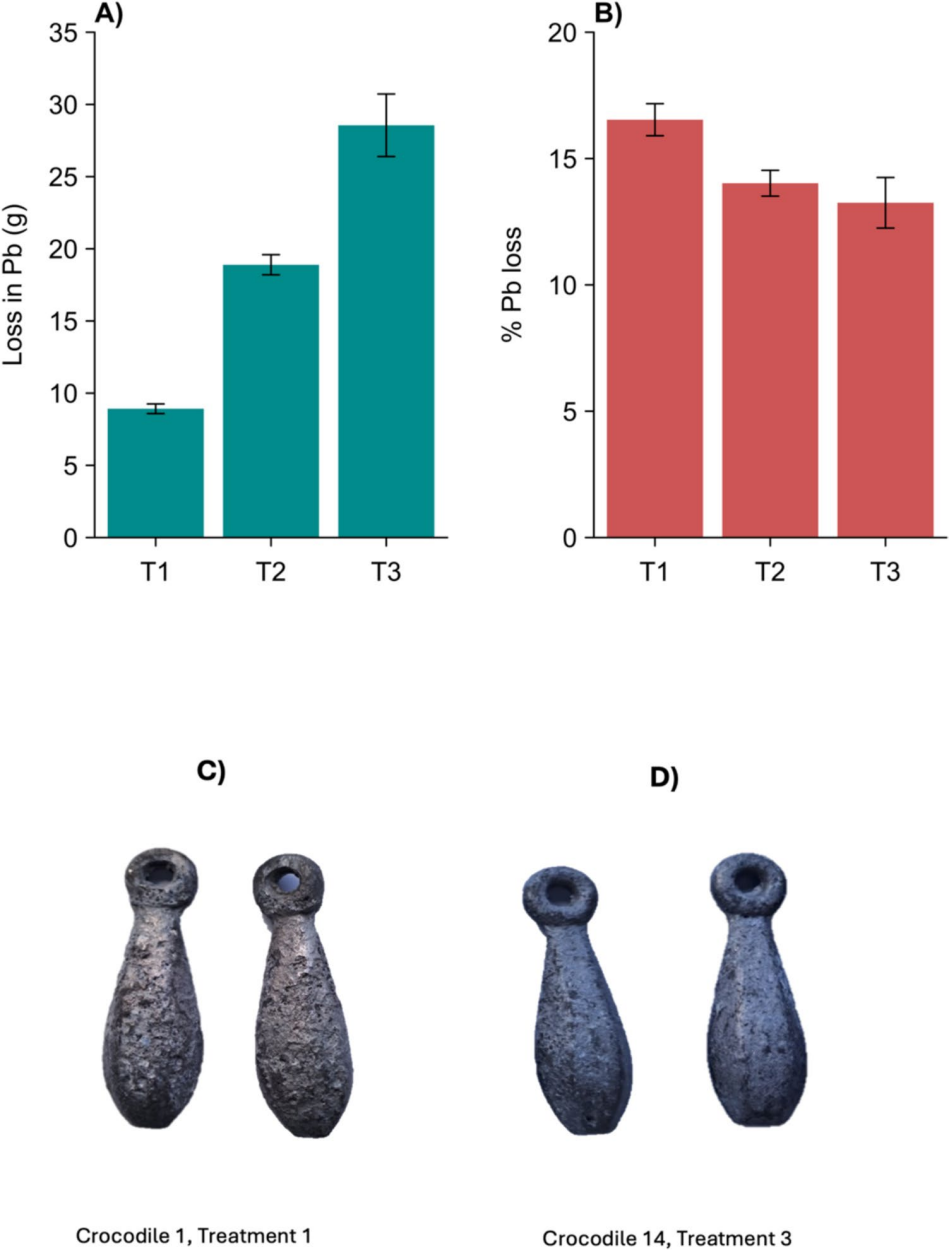
**A** Mean loss in lead (Pb) weight after 48 weeks, and **B** mean percentage Pb loss per treatment group after 48 weeks. Treatments (T): T1 = 2 × 1 oz sinkers (~ 54 g Pb, *n* = 5); T2 = 5 × 1 oz sinkers (~ 135 g Pb, *n* = 3); T3 = 8 × 1 oz sinkers (~ 215 g Pb, *n* = 6). Results are presented as mean values, with error bars representing the standard error of the mean (SEM) **C** photograph of Pb sinkers after removal from the stomach of crocodile 1 (Treatment 1: 2 × 1 oz sinkers, ~ 54 g Pb) 48 weeks after ingestion, **D** photograph of Pb sinkers after removal from the stomach of crocodile 14 (Treatment 3: 8 × 1 oz sinkers, ~ 215 g Pb) 48 weeks after ingestion

Loss in Pb weight varied in proportion to Pb dosage (Fig. 7A and B), with significant differences in percentage weight loss across all treatment groups (*χ*^2^ = 6.1, *df* = 2, *p* = 0.047) and this erosion on the Pb weights was visible to the naked eye (Fig. 7C and D). For the crocodiles administered with two Pb weights (treatment 1), the percentage reduction in Pb mass averaged 16.5%. At this rate, complete dissolution would occur in ~ 5.8 years. A mean percentage reduction of 13.2% was recorded in crocodiles with eight Pb sinkers (treatment 3). At this rate, complete dissolution would take place in ~ 7.3 years.

### Body weight and morphometrics

**Fig. 8:**
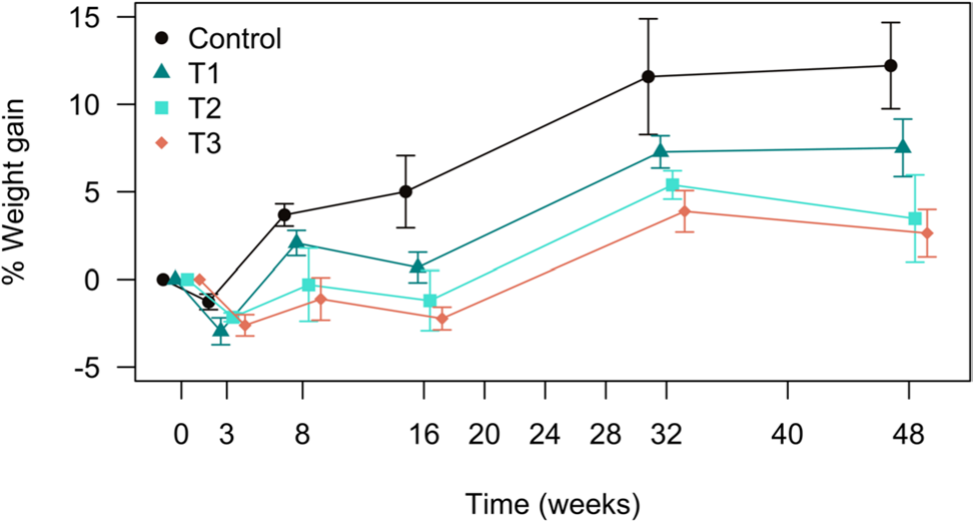
Mean percentage weight change in crocodiles exposed to varying lead (Pb) dosages over the duration of the study. Treatments (T): Control = no Pb exposure (*n* = 4); T1 = 2 × 1 oz sinkers (~ 54 g Pb, *n* = 5); T2 = 5 × 1 oz sinkers (~ 135 g Pb, *n* = 3); T3 = 8 × 1 oz sinkers (~ 215 g Pb, *n* = 6). Results are presented as mean values, with error bars representing the standard error of the mean (SEM). Negative week values represent baseline weight measurements collected before the crocodiles were fed lead (Pb) sinkers at Week 0

Throughout the 48-week experimental period, none of the crocodiles displayed any obvious clinical signs typically associated with Pb toxicity. Despite all individuals appearing physically healthy, variations in growth were noted across the treatment groups (Fig. 8). The control group of crocodiles gained weight consistently throughout the study, with a total body mass increase of 12.2%. In contrast, crocodiles in treatment group 1 gained less weight (7.5%), and this reduction was even more pronounced in treatment groups 2 and 3, which only exhibited body mass increases of 3.5% and 2.6%, respectively. However, a statistically significant difference in body mass increase between the control group and Pb-exposed crocodiles was only observed for treatment group 3 (*p* = 0.010).

Although growth differed between groups over the study period, morphometric measurements showed few significant variations overall. Specifically, total length growth did not differ significantly (Kruskal–Wallis test statistic: 2.19; *p* = 0.53) between the treatment groups and the control group over the study duration. Similarly, tail girth showed no significant change from the start to the end of the study (t-statistic: − 0.28; *p* = 0.79). A significant difference was only observed in neck girth, where treatment group 3 exhibited significantly less growth compared to the control group (mean difference =  − 3.12 cm; *p* = 0.011).

Snout–vent length (SVL) did not differ significantly between week 0 and week 48 (Shapiro–Wilk: W = 0.904, *p* = 0.067; paired *t* test: *t*(17) = 1.82, *p* = 0.086; week 0: 97.7 ± 5.6; week 48: 96.9 ± 4.9). Mid-body girth (MBG) also showed no significant difference between week 0 and week 48 (Shapiro–Wilk: W = 0.983, *p* = 0.979; paired *t* test: *t*(17) = 1.02, *p* = 0.324; week 0: 68.9 ± 4.4; week 48: 68.3 ± 4.4).

**Fig. 9:**
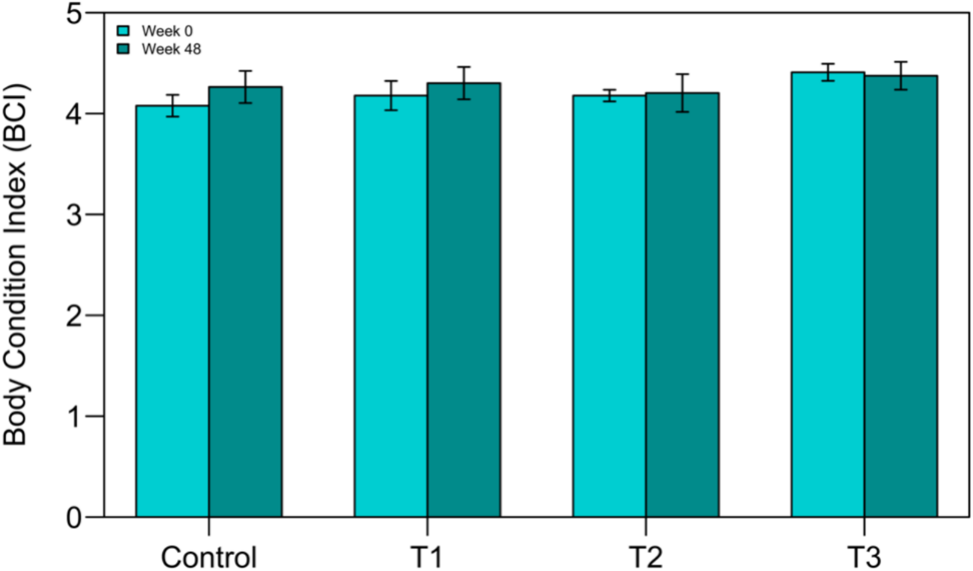
Body condition index (BCI) of crocodiles at the start (week 0) and end (week 48) of the exposure period across all treatment groups. BCI is a measure of the animal’s health and body condition. Treatments: Control = no Pb exposure; Treatment 1 = 2 × 1 oz sinkers (~ 54 g Pb); Treatment 2 = 5 × 1 oz sinkers (~ 135 g Pb); Treatment 3 = 8 × 1 oz sinkers (~ 215 g Pb). Results are presented as mean values, with error bars representing the standard error of the mean (SEM)

The control group showed a statistically significant increase in BCI (*p* = 0.005) over the duration of the study, indicating an improvement in body condition of individuals not exposed to Pb. In contrast, no statistically significant changes in BCI were observed for crocodiles in any of the treatment groups (Fig. 9).

## Discussion

### Blood lead concentrations

This study represents the longest Pb dosage experiment ever performed on a crocodilian. Despite exposing crocodiles to large quantities of Pb (up to 215 g), none of the individuals died over the 48-week duration of the study or displayed any obvious clinical signs typically associated with Pb toxicity. This finding indicates that crocodiles exhibit remarkable tolerance to extreme BPb concentrations.

Our results show that the onset of dissolution of ingested sinkers is rapid and Pb is quickly absorbed into the blood. In our study, BPb concentrations increased between 40-fold in the lowest dosage group to almost 100-fold in the highest dosage group during the first week of exposure. Although BPb concentrations initially appeared to stabilise 3–4 weeks into the experiment, concentrations continued to rise over the duration of the study, with maximum BPb concentrations recorded at 48 weeks in all treatment groups. During the experiment, BPb reached concentrations that have never previously been measured in crocodilians. The highest BPb concentration ever recorded in a wild crocodile is 13,100 ng/ml, measured in an adult male *C. niloticus* from Lake St Lucia, South Africa (Humphries et al. 2022). By the end of the study, all treatment crocodiles greatly exceeded this value. The highest BPb concentration of 82,800 ng/ml measured in crocodile 15 (treatment group 3) at 48 weeks is to our knowledge the highest BPb ever recorded in a living vertebrate.

Increases in BPb concentrations across the three treatment groups varied in relation to dosage. It is therefore not surprising that the BPb concentrations measured in this study were considerably higher than those reported by Hammerton et al. (2003) in their experiment with *C. porosus*, given the considerably larger Pb quantities used in our investigation. Hammerton et al. (2003) used a maximum Pb dose of 3.76 g (administered as Pb shot), whereas our treatment group 1 (54 g) and treatment group 3 (215 g) received doses ~ 14 and ~ 57 times greater, respectively. At the conclusion of their 20-week study, Hammerton et al. (2003) recorded a peak BPb concentration of 5140 ng/ml. In contrast, after 20 weeks in our experiment, the mean BPb values for the three treatment groups were 14,000 ± 980 ng/ml for treatment 1 (T1), 23,400 ± 5250 ng/ml for treatment 2 (T2), and 19,700 ± 2000 ng/ml for treatment 3 (T3). Hammerton et al. (2003) suggested that BPb reached a steady-state equilibrium after 20 days for crocodiles receiving 1.93 g of Pb (5 Pb shot) and after 85 days for those receiving 3.76 g (10 Pb shot). Given the 20-week duration of their study, it remains uncertain if the plateau in BPb concentrations they observed truly represented steady-state equilibrium. In contrast, an equilibrium state was not achieved within the 48-week period of this study.

We recorded some variation in BPb concentrations among crocodiles receiving equivalent lead doses. This individual variability may be partly explained by differences in how much food each crocodile consumed. Although all individuals were presented with the same feeding opportunities, the farm’s practice of feeding crocodiles until they were satiated likely led to variations in the quantity of food consumed among individuals. This difference in food intake could, in turn, affect Pb absorption in the stomach during digestion, potentially explaining some of the observed variations in BPb within treatment groups. Lead absorption is likely also influenced by temperature. Like other ectotherms, the metabolism of crocodilians slows down during winter (Grigg and Kirshner 2015), which may reduce Pb absorption from the stomach into the blood. The lower metabolism associated with cooler temperatures also affects appetite, leading to less frequent feeding. This pattern is supported by our temperatures recordings, which show a decline in water temperature between weeks 16 and 28 corresponding with lower Pb uptake across treatment groups. Similarly, between weeks 40 and 48, we observed an increase in Pb uptake corresponding with rising temperature. This suggests that temperature likely influenced the rate of Pb absorption by affecting feeding and metabolic rate.

Based on the percentage reduction in the weight of the lead shot over the 48-week period, we estimated that it would take between 5.8 and 7.3 years for complete dissolution of Pb fishing weights in the stomach of the crocodiles used in this study. However, erosion rates are likely to vary considerably depending on the size and shape of the Pb object. We selected 2-oz fishing sinkers for our experiment because of their popularity among anglers at Lake St Lucia. In their experimental study, Hammerton et al. (2003) estimated that the lead shot (4.1 mm diameter) they used would take 1.5—3 years to fully dissolve. In natural settings, the presence of other gastroliths in the stomach of a crocodile may potentially accelerate dissolution through mechanical abrasion. Despite these potential influencing factors, our findings suggest that an ingested fishing sinker is likely to remain in a crocodile’s stomach for multiple years. This prolonged retention has implications for understanding Pb exposure in wild crocodile populations. The suggestion by Humphries et al. (2022) that crocodiles at St Lucia could have been subjected to long-term Pb exposure as a result of sinkers retained in their stomach for several years, or even decades, is supported by the erosion rates calculated in this study.

### Tolerance to Pb and clinical signs of toxicity

All treatment crocodiles in this study reached BPb concentrations that far exceeded established thresholds for severe clinical poisoning in birds and mammals. In birds, BPb concentrations between 500 and 1000 ng/ml are associated with clinical poisoning (Pain 1996), whereas in mammals, BPb concentrations > 600‒800 ng/ml are often associated with clinical signs of toxicity, including anaemia, neurological impairment, and reproductive failure (Ma 1996). Our findings are consistent with recent studies on lizards (Blanchette et al. 2025; Moore et al. 2025), suggesting that reptiles are relatively tolerant to the acute effects of high concentrations of Pb.

Although none of the crocodiles in this study displayed obvious signs of Pb poisoning over the duration of the study, increases in BPb in our experimental animals were generally associated with lower PCVs. Lead toxicity is known to reduce PCV and can cause anaemia in birds and mammals by impairing both erythrocyte production and survival. Lead has potent inhibitory effects on key enzymes integral in the production of haeme, a molecular component of haemoglobin (Warren et al. 1998). The most well known of these is δ- aminolevulinic acid dehydrogenase (ALAD; Scinicariello et al. 2007). Additionally, Pb disrupts cellular membrane integrity through the production of reactive radicals and interference with the enzymes that maintain membrane stability (Wani et al. 2015), both of which lead to cellular damage and destruction.

In reptile species, temperature has also been shown to influence PCV, with higher temperatures being associated with higher PCV values (Stacy et al. 2011). Mean PCVs in control crocodiles varied between 25 and 32% throughout the study. Initially high, PCVs gradually decreased between weeks 16 and week 40 (March to August), likely due to declining environmental temperatures in autumn and winter months. Subsequently, an increase in PCV was observed at week 48 (October), consistent with warmer temperatures in spring. Packed cell volumes in crocodiles from all three treatment groups fell below our calculated reference range, indicating anaemia. This was first observed at week eight and persisted until the end of the study. However, none of the dosed crocodiles exhibited any noticeable clinical signs typically associated with anaemia. The most obvious signs are associated with a decrease in capacity of the blood to deliver oxygen to tissues (Saggese 2009), and include weakness, dyspnoea, and exercise intolerance. However, these are often difficult to assess in reptiles due to their generally low activity levels, a factor further compounded in this study by the limited space of their enclosures.

It is somewhat surprising the dosed crocodiles did not develop more severe anaemia during this study. Blood smear analysis at weeks eight and 48 indicated a high level of RBC regeneration in Pb-exposed crocodiles. This suggests that increased RBC production may be compensating for the loss of RBCs due to destruction. This finding is particularly interesting given lead’s established negative effect on haeme synthesis. The increased frequency of basophilic stippling observed in blood smears from the treatment groups is also interesting. These inclusions in the cytoplasm of the RBC are due to aggregates of ribosomal RNA (Samour and Hart 2021). In Pb poisoning, the functioning of the enzyme 5′ nucleotidase is disrupted, which normally degrades the residual RNA (Warang et al. 2017). However, it is also not uncommon to see mild basophilic stippling during normal RBC regeneration in healthy individuals (Stacy et al. 2011). Thus, it is difficult to attribute more frequent incidences of basophilic stippling in treatment crocodiles to the direct effects of Pb or to the increased level of RBC.

Humphries et al. (2022) documented anaemia in Lake St Lucia crocodiles with BPb exceeding 6000 ng/ml. These individuals exhibited considerably lower PCV (4.6–10.8%), two to four times lower than mean concentrations measured in unaffected wild crocodiles. Notably, one crocodile with a PCV of 5.5% exhibited obvious signs of anaemia, including lethargy and unusual paleness. Despite much higher BPb concentrations in our study, PCVs did not decrease to the low levels reported in wild crocodiles at St Lucia. This discrepancy likely reflects differences in exposure duration. It is possible that a crocodile’s ability to regenerate RBCs may decline with prolonged Pb exposure.

While the underlying reasons are speculative, one possibility involves impacts on bone marrow. It is well known that lead is stored in bone during chronic exposure, potentially increasing the likelihood of Pb affecting RBC precursors in the bone marrow and leading to a non-regenerative anaemia. It has been suggested that heavy metal exposure may be associated with aplastic or non-regenerative anaemias in humans (Jain et al. 2023). Additionally, many diseases affecting the kidney and liver have been associated with non-regenerative anaemia (Divers 2000; Saggese 2009). Given the propensity of Pb to accumulate in these organs, it is possible that lead poisoning may contribute to non-regenerative anaemia through this mechanism. It has been suggested that many reptiles will only begin to manifest clinical signs of anaemia when they have developed a non-regenerative anaemia that is moderate to severe (Saggese 2009).

It is evident that a study of longer duration is necessary to fully understand the chronic effects of lead poisoning in crocodilians. Although our 48-week experiment represents the longest Pb exposure study in a reptilian species to our knowledge, it is likely insufficient to reveal the true long-term consequences. Currently, our best insights into potential long-term effects come from studies involving wild animals. Humphries et al. (2022) considered tooth loss in crocodiles at Lake St Lucia to be a sign of long-term environmental exposure to Pb. Given the ability of Pb to substitute for calcium in hydroxyapatite, the main mineral component of bone and teeth, its accumulation in dental tissues can compromise their structural integrity. In affected crocodiles, lost teeth are seemingly not replaced, suggesting that Pb exposure causes prolonged damage to the underlying bone tissue or dental lamina which impedes the development of new teeth. Future analysis of samples collected from the crocodiles in this study will provide insight into the rate at which Pb accumulates in the teeth and jaw bone.

Little is known about the effect of Pb exposure on growth and development. In this study, Pb exposure appeared to influence weight gain over the duration of the study, although the precise mechanisms remain unclear and likely involve multiple factors. No clear morphometric differences (TL, SVL, TG, and MBG) were observed between treatment groups over the duration of the study. Body condition index improved in control animals but not in Pb-exposed groups, suggesting that Pb primarily influenced weight gain rather than structural growth. Lead exposure is known to affect appetite, but without detailed data on food offered and consumed, we cannot draw conclusions about its impact on food intake. With long-term exposure, Pb accumulates in various soft tissues, particularly the liver and kidneys. It is therefore possible that Pb poisoning in these animals may be causing protein loss through various aetiologies affecting these organs. Future biochemical and histopathological analyses will investigate potential organ disease in the study population.

### Fate of Pb

To our knowledge, no published studies have directly investigated urine as a potential pathway of Pb excretion in crocodiles. The results of this study show that excretion of Pb via urine is insignificant compared to the body burden. Although UPb concentrations increased sharply during the first few weeks following exposure, concentrations remained low (typically < 10 ng/ml) even in the high dosage group. Urinary Pb concentration declined over the following months even as BPb concentrations increased substantially. This suggests that urine is not an important route through which crocodiles rid themselves of Pb.

Lead entering the bloodstream likely accumulates primarily in bone and, to a lesser extent, in organs such as the liver and kidney. While this study did not directly examine the effects of Pb accumulation on physiological health, future planned analyses, such as histopathology and biochemical assessments, will provide insight into the potential impacts of Pb on organ function and overall health.

## Conclusions

Sub-adult Nile crocodiles exhibited a remarkable tolerance to exceptionally high lead (Pb) exposure during our 48-week study. Despite reaching blood lead (BPb) concentrations that far exceeded established thresholds for severe clinical poisoning in birds and mammals, crocodiles remained in seemingly healthy condition. This observation aligns with findings from prior crocodilian studies. However, despite the absence of overt clinical signs associated with Pb toxicity, we found that increases in BPb were generally associated with lower parked cell volumes (PCVs). Across all treatment groups, PCVs fell below the calculated reference range by week eight and remained suppressed until the study’s conclusion, indicating the development of anaemia. Dosed crocodiles demonstrated a notable capacity for red blood cells (RBC) regeneration, which may partly explain their resilience to the acute toxic effects of Pb. Exposure to Pb did not significantly impact the body condition of the crocodiles but exerted a discernible influence on body mass, with treated crocodiles exhibiting substantially reduced weight gain throughout the study period. This observation suggests that Pb may affect appetite or induce protein loss, potentially associated with organ dysfunction.

We estimated that it would take between 5.8 and 7.3 years for the Pb fishing weights used in this experimental study to completely dissolve within the stomach of a crocodile. This has significant implications for wild crocodile populations, which are likely to experience Pb exposure over considerably longer durations. Consequently, while this study represents the longest investigation of Pb exposure in a reptilian species to date, the 48-week timeframe is likely insufficient to fully capture the long-term physiological consequences observed in wild populations. Finally, the potential for sub-clinical health effects arising from Pb exposure in our studied crocodiles warrants consideration. Future biochemical and histopathological analyses are planned to provide a more comprehensive understanding of the physiological consequences associated with Pb exposure in crocodilians.
